# Massive Renal Cyst Displacing Intra-Abdominal Structures

**DOI:** 10.31486/toj.23.0036

**Published:** 2023

**Authors:** Khalid M. G. Mohammed, Ayaa Zarm, Juan Carlos Q. Velez, Muner M. B. Mohamed

**Affiliations:** ^1^ School of Medicine, Tanta University, Tanta, Egypt; ^2^ Department of Nephrology, Ochsner Clinic Foundation, New Orleans, LA; ^3^ The University of Queensland Medical School, Ochsner Clinical School, New Orleans, LA

**Keywords:** *Cysts*, *flank pain*, *hyperkalemia*

## Abstract

**Background:** Simple renal cysts typically produce no symptoms or signs and are usually detected incidentally on imaging studies for unrelated causes. Massive renal cysts are very rare.

**Case Report:** A 77-year-old female with preexisting chronic kidney disease presented to our hospital for evaluation of hyperkalemia, abdominal distension, and right flank pain. Upon arrival, her vital signs and physical examination were normal. Laboratory data were pertinent for a serum creatinine of 4.8 mg/dL (6 months prior to presentation, serum creatinine was 1.5 mg/dL, and 1 month after discharge, it was 4.6 mg/dL), and hyperkalemia of 6.0 mmol/L. Computed tomography revealed a massive right renal cyst measuring 22 × 11 × 17.5 cm and displacing the intra-abdominal structures. Because of her symptoms, the patient was evaluated by urology for surgical management. The patient refused invasive procedures and chose pain control and monitoring.

**Conclusion:** Noninvasive treatment options for a massive simple renal cyst are limited. Symptomatic treatment and monitoring the cyst size on a regular basis might be helpful for patients who refuse invasive treatment.

## INTRODUCTION

Renal cysts are common in adults, and the incidence increases as individuals age.^1^ Although usually asymptomatic, renal cysts may occasionally present with pain, infection, hypertension, or mass effect.^2^ We report the case of a 77-year-old female with a massive renal cyst.

## CASE REPORT

A 77-year-old female was admitted to our hospital for hyperkalemia and right flank pain. She had a medical history of chronic kidney disease, hypertension, type 2 diabetes mellitus, remote partial left nephrectomy secondary to benign oncocytoma, bariatric surgery, peripheral arterial disease, atrial flutter, and breast cancer. Her medications included rivaroxaban, amiodarone, anastrozole, atorvastatin, cholecalciferol, gabapentin, pantoprazole, and lisinopril. The day before admission, she was seen by her family doctor for right flank pain; routine blood work and urinalysis were ordered. The following day, her family doctor asked her to go to the emergency department because her blood work showed hyperkalemia and elevated serum creatinine (sCr). She reported worsening right flank pain of 4 days’ duration. The pain was dull and associated with nausea and early satiety. The patient did not report fever, chills, dyspnea, vomiting, hematuria, or voiding difficulty. Physical examination revealed distended abdomen without any tenderness and pitting edema of the bilateral lower extremities. The patient's temperature was 98.6 °F (37 °C), blood pressure was 119/59 mm Hg, heart rate was 70 beats/min, and respiratory rate was 17 breaths/min. The remainder of the examination was normal.

The patient was admitted for hyperkalemia of 6.0 mmol/L and was noted to have elevated sCr of 4.8 mg/dL; her sCr value 6 months prior to admission was 1.5 mg/dL. Other routine hematology, biochemistry, and urinalysis values were within normal limits except for hemoglobin of 7.7 g/dL, bicarbonate of 11 mmol/L, blood urea nitrogen of 86 mg/dL, phosphorus 6.3 mg/dL, parathyroid hormone of 799.1 pg/mL, and magnesium of 1.3 mg/dL (Table 1). No obvious reason suggested a diagnosis of acute kidney injury. The patient had not seen a nephrologist as an outpatient. The hyperkalemia was medically treated with regular insulin 8.35 units once, albuterol nebulizer 10 mg once, and sodium zirconium cyclosilicate 10 g 3 times daily for 6 doses.

**Table 1.** Admission and Last-Reported Preadmission Laboratory Data

Parameter	Reference Range	Laboratory Results on Admission, March 6, 2023	Prior Laboratory Results, September 29, 2022
**Clinical chemistry**			
Sodium, mmol/L	136-145	139	139
Potassium, mmol/L	3.5-5.1	6.0	4.1
Chloride, mmol/L	95-110	111	107
Bicarbonate, mmol/L	23-29	11	20
Anion gap, mmol/L	5-15	17	12
Blood urea nitrogen, mg/dL	6-20	86	45
Serum creatinine, mg/dL	0.5-1.4	4.8	1.5
Estimated glomerular filtration rate*,* mL/min/1.73 m^2^	>60	8.8	35.7
Calcium, mg/dL	8.7-10.5	8.8	10.3
Glucose, mg/dL	70-110	89	87
Phosphorus, mg/dL	2.7-4.5	6.3	
Magnesium, mg/dL	1.6-2.6	1.3	
Alkaline phosphatase, U/L	55-135	76	89
Protein total, g/dL	6.0-8.4	6.9	6.3
Albumin, g/dL	3.5-5.2	2.9	2.8
Bilirubin total, mg/dL	0.1-1.0	0.3	0.4
Aspartate transaminase, U/L	10-40	12	21
Alanine transaminase, U/L	10-44	10	14
C-reactive protein, mg/L	0.0-8.2	13	149.5
**Complete blood count**			
Hemoglobin, g/dL	14.0-18.0	7.7	10.3
Platelets, K/μL	150-350	247	170
White cell count, K/μL	3.90-12.70	5.7	6.4
**Iron/anemia profile**			
Iron, μg/dL	45-160	47	19
Total iron binding capacity, μg/dL	250-450	260	333
Saturated iron, %	20-50	18	6
Transferrin, mg/dL	200-375	176	225
Parathyroid hormone, intact, pg/mL	9.0-77.0	799.1	
**Urine**			
Color	Yellow, straw, amber	Yellow	Yellow
Appearance	Clear	Clear	Clear
Specific gravity	1.005-1.030	1.010	1.010
pH	5.0-8.0	5.0	6.0
Glucose	Negative	Negative	Negative
Protein	Negative	Negative	Negative
Ketones	Negative	Negative	Negative
Occult blood	Negative	Negative	Negative
Nitrite	Negative	Negative	Negative
Bilirubin	Negative	1+	Negative
Leukocytes, hpf	0-4	0	
Red blood cells, hpf	0-5	6	
White blood cells, hpf	None-occasional	Rare	
Bacteria, hpf	0.00-0.20	Undetectable	
Protein/creatinine ratio	0.00-0.20	0.19	0.17

**Figure 1.**
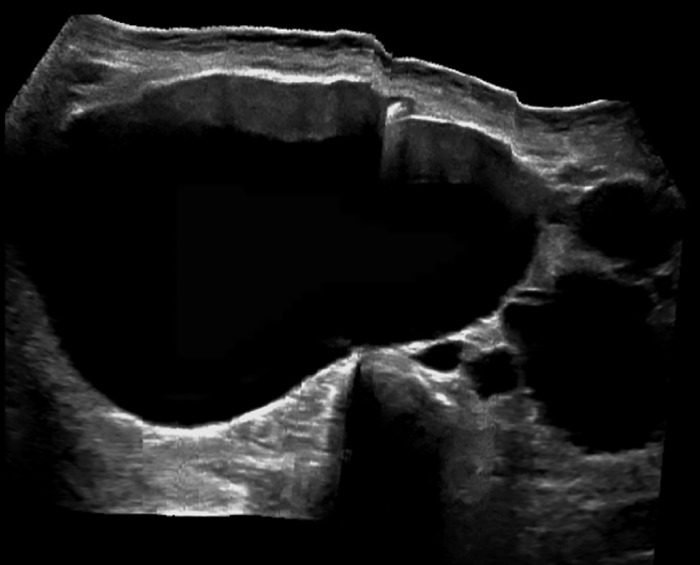
Ultrasonography of right kidney revealing a massive simple renal cyst.

**Figure 2.**
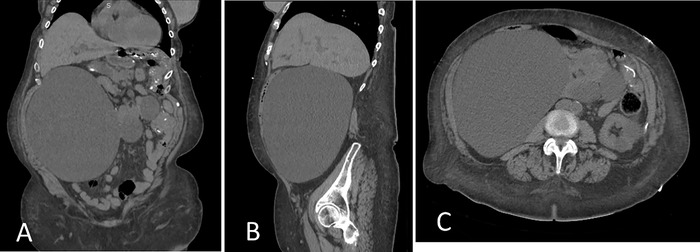
Computed tomography of the abdomen revealing a massive simple cyst on (A) coronal, (B) sagittal, and (C) axial views.

Ultrasonographic examination of the kidneys revealed a massive right simple cyst approximately 22.6 × 10.8 × 17.5 cm (Figure 1). Computed tomography of the abdomen revealed numerous simple cysts involving the right kidney, the largest measuring 22 × 11 × 17.5 cm and displacing surrounding structures (Figures 2A and 2B). The right kidney was displaced toward the midline (Figure 2C). Postoperative changes of partial left nephrectomy were noted. Multiple simple cysts were also noted in the left kidney, the largest in the superior pole and measuring 4.2 cm. No evidence of hydronephrosis was seen. The ureters were unremarkable. The pancreas was displaced anteriorly. Urology evaluated the patient for surgical resection. After options were discussed (observation, aspiration, decortication), the patient opted out of surgical procedures because of the fear of possibly losing her kidney and being placed on dialysis. Pain and discomfort were managed with acetaminophen. One month after discharge, the patient was seen at our nephrology clinic. Her sCr was 4.5 mg/dL, indicating that the high sCr seen on admission was most likely a progression of the patient's chronic kidney disease.

## DISCUSSION

Simple renal cysts are the most common acquired cystic disease in adults, especially in the elderly, accounting for approximately 65% to 70% of all kidney masses.^3^ In healthy individuals, the prevalence of simple cysts has been reported to be 10.7%.^4^ Most of the time, renal cysts are asymptomatic and detected incidentally during imaging studies performed for other reasons. The cysts can be solitary or multiple and can involve both kidneys.^5^ In a study of 1,948 potential kidney donors, after adjusting for age and sex, renal cysts ≥5 mm were associated with high blood pressure, higher albumin excretion, and hyperfiltration.^6^ In a longitudinal study, 45 patients with renal cysts were followed for 6 years. Most of the renal cysts increased in size and number, with higher growth rates of 3.94 mm/year in patients younger than 50 years and 1.84 mm/year in patients 50 years and older.^7^ Simple cysts typically are asymptomatic. Rarely, simple cysts associated with rupture causing hematuria grow to large sizes that cause abdominal pain, discomfort, mass effect on surrounding structures, and/or infection.

Massive renal cysts are very rare. To our knowledge, only 5 cases of renal cysts >15 cm have been reported, and all cases were treated with intervention (Table 2).^8-12^ Most cysts do not require treatment unless they become infected; in such cases, antibiotics are needed. If the cyst size increases to the point of causing a pressure effect on other organs and pain, the treatment options are either percutaneous drainage and sclerotherapy using ethanol or laparoscopic excision.^13^ In our case, the patient refused invasive intervention.

**Table 2.** Reported Cases of Simple Renal Cysts >15 cm

Study	Patient Age, years/Sex	Cyst Size, cm	Presentation	Treatment
Giannakopoulos et al, 2005^8^	82/M	36 × 19	Right flank pain, high blood pressure 210/125 mm Hg	Percutaneous drainage
Ahallal et al, 2009^9^	25/F	17 × 20	Right flank pain, high blood pressure 190/125 mm Hg	Laparoscopic excision
Rehman et al, 2014^10^	27/F	24 × 18	Abdominal pain, abdominal distension	Deroofing and intraperitoneal marsupialization of the cyst
Riyach et al, 2014^11^	75/M	35 × 32 × 22	Suprapubic pain, abdominal distension, mimicking ascites	Percutaneous drainage
Fernández-de la Varga et al, 2022^12^	81/F	16	Large bowel obstruction	Percutaneous drainage

F, female; M, male.

No evidence-based guidelines are available for managing a massive simple renal cyst, especially in patients who refuse surgical intervention. We anticipate a challenge in monitoring the growth of the cyst, symptomatology, and possible complications in the future. For our patient, the plan is to monitor the size of the cyst by performing renal ultrasound every 6 months and educating the patient about the symptoms and signs of associated complications.

## CONCLUSION

Massive simple renal cysts are rare. Therapy may be required for complications such as infection, rupture, and mass effect, including invasive procedures. Noninvasive management is limited to monitoring on a regular basis, controlling the pain, and treating associated infection.

## Notes

*This article meets the Accreditation Council for Graduate Medical Education and the American Board of Medical Specialties Maintenance of Certification competencies for Patient Care and Medical Knowledge*.
